# Metagenome analyses of corroded concrete wastewater pipe biofilms reveal a complex microbial system

**DOI:** 10.1186/1471-2180-12-122

**Published:** 2012-06-22

**Authors:** Vicente Gomez-Alvarez, Randy P Revetta, Jorge W Santo Domingo

**Affiliations:** 1 U.S. Environmental Protection Agency, Office of Research and Development, Cincinnati, OH, 45268, USA

## Abstract

**Background:**

Concrete corrosion of wastewater collection systems is a significant cause of deterioration and premature collapse. Failure to adequately address the deteriorating infrastructure networks threatens our environment, public health, and safety. Analysis of whole-metagenome pyrosequencing data and 16S rRNA gene clone libraries was used to determine microbial composition and functional genes associated with biomass harvested from crown (top) and invert (bottom) sections of a corroded wastewater pipe.

**Results:**

Taxonomic and functional analysis demonstrated that approximately 90% of the total diversity was associated with the phyla Actinobacteria, Bacteroidetes, Firmicutes and Proteobacteria. The top (TP) and bottom pipe (BP) communities were different in composition, with some of the differences attributed to the abundance of sulfide-oxidizing and sulfate-reducing bacteria. Additionally, human fecal bacteria were more abundant in the BP communities. Among the functional categories, proteins involved in sulfur and nitrogen metabolism showed the most significant differences between biofilms. There was also an enrichment of genes associated with heavy metal resistance, virulence (protein secretion systems) and stress response in the TP biofilm, while a higher number of genes related to motility and chemotaxis were identified in the BP biofilm. Both biofilms contain a high number of genes associated with resistance to antibiotics and toxic compounds subsystems.

**Conclusions:**

The function potential of wastewater biofilms was highly diverse with level of COG diversity similar to that described for soil. On the basis of the metagenomic data, some factors that may contribute to niche differentiation were pH, aerobic conditions and availability of substrate, such as nitrogen and sulfur. The results from this study will help us better understand the genetic network and functional capability of microbial members of wastewater concrete biofilms.

## Background

Concrete corrosion of wastewater collection systems is a significant cause of deterioration and premature failure. In the U.S., costs associated with maintaining an estimated 800,000 miles of wastewater collection infrastructure are approximately $4.5 billion per year [1]. Many systems may be beyond their design life and must be replaced because they cannot be rehabilitated [2]. Failure to adequately address the deteriorating infrastructure networks threatens our environment, public health, and safety. In wastewater collection systems microbial-induced concrete corrosion (MICC) may occur in areas under higher concentrations of hydrogen sulfide (H_2_S) [3-5]. The primary source of sulfur is sulfate (SO_4_^2-^) which can be reduced by sulfate-reducing bacteria (SRB) to hydrogen sulfide (H_2_S) under anaerobic conditions. H_2_S is transferred across the air-water interface to the sewer atmosphere where chemoautotrophic bacteria on the pipe surface, including sulfide-oxidizing bacteria (SOB), convert the H_2_S to biogenic sulfuric acid (H_2_SO_4_). Biogenic sulfuric acid (H_2_SO_4_) can be generated by various microbial species [6-9].

While many of the microorganisms and general mechanism involved in MICC has been known for decades, and recent studies using molecular-based approaches have more accurately described the microbial ecology of these engineered systems [6,8,9], a better understanding of the metabolic processes and functional capabilities is needed to develop new approaches to mitigate MICC and its associated effects. The objective of this study was to characterize the microbial community of concrete wastewater biofilms and their functional capability based on molecular analyses of metagenome libraries and to compare it with 16S rRNA gene sequences from previously generated clone libraries [7-11]. Specifically, we sampled biofilms from two sections of a severely corroded concrete wastewater pipe to obtain a better understanding of microbial community colonization processes and mechanisms of concrete deterioration. To our knowledge this is the first published report utilizing metagenomics to elucidate microbial community functional capabilities involved in MICC in wastewater collection systems.

## Methods

### Sampling and extraction of total DNA from biofilms

Biofilm samples were collected from two sections of a corroded concrete sewer pipe located in the Cincinnati metropolitan area. The excavated pipe was installed in 1949 and exposed to residential waste. Biomass was removed from the crown (top section of the pipe, TP) and invert (bottom, BP) sections using a sterile metal spatula by scraping approximately 4 cm^2^ surface area of each material. Biomass was then transferred to sterile tubes and stored at −20°C. Total DNA was extracted using UltraClean Soil DNA kit following the manufacturer’s instructions (MoBio Laboratories Inc., Solana Beach, CA) and used as a template for the generation of pyrosequencing metagenome libraries.

### 16S rRNA gene sequence analyses

Sequences from Bacteroidetes (*n*=236), sulfate reducing (*n*=56) and sulfur oxidizing (*n*=164) bacteria obtained from a previous study [11] were used to develop phylogenetic trees. Briefly, 16S rRNA gene primers 8F and 787R were used to generate community PCR products, which were then cloned using TOPO TA vectors. Clones were sequenced in both directions and assembled using Sequencher software (Gene Codes Corp, Ann Arbor, MI). Sequences were assigned to specific bacterial groups using MOTHUR v1.19.2 (http://www.mothur.org) with 97% sequence identity as the cut off point for each Operational Taxonomic Unit (OTU). Phylogenetic trees were constructed from the alignments based on the Maximum Likelihood method and calculated using Tamura-Nei model [12]. MEGA v5.03 [13] was used to build trees using 100 replicates to develop bootstrap confidence values. The Classifier tool of the Ribosomal Database Project II release 10.26 [14] and BLASTn [15] were used to classify and identify the nearest neighbors.

### Cluster analysis of wastewater concrete biofilms

Cluster analysis based on the transformed (log[x+1]) relative abundance data was used to compare communities associated with different wastewater concrete biofilms. First, we estimated the taxonomic distribution at the genus level of each microbial community from 16S rRNA gene pyrosequences generated in this study and Sanger-chemistry 16S rRNA gene sequences generated in previous studies [7-10]. This information was used to generate Bray-Curtis similarity coefficients of the transformed data using the software PAST v2.03 [16]. This estimator compares the structures by accounting for the abundance distributions of attributes (e.g. species). Dendrograms indicating relationship of biofilms generated by comparing similarity coefficients estimates among sample sites were calculated using the UPGMA method with the software MEGA v5.03 [13].

### Metagenomic studies

Pyrosequencing was performed using the 454 Life Sciences GS-FLX Titanium® platform. Prior to sequence analysis we implemented a dereplication pipeline (http://microbiomes.msu.edu/replicates) to identify and remove clusters of artificially replicated sequences, i.e. reads that began at the same position but varied in length or contained a sequencing discrepancy [17]. Filter parameters included a cutoff value of 0.9, no length difference requirement and an initial base pair match of 3 base pairs. Metagenome sequence data (i.e. singleton reads) were processed using two fully automated open source systems: (1) the MG-RAST v3.0 pipeline (http://metagenomics.anl.gov) [18] and (2) the Rapid Analysis of Multiple Metagenomes with a Clustering and Annotation Pipeline (RAMMCAP) [19], available from the Community Cyberinfrastructure for Advanced Microbial Ecology Research and Analysis (CAMERA, http://camera.calit2.net). The analysis included phylogenetic comparisons and functional annotations. All analyses were performed with an expected e-value cutoff of 1e^-05^ without preprocessing filtering. The metagenomes generated in this paper are freely available from the SEED platform (Projects: 4470638.3 and 4470639.3). Taxonomic relationships between metagenomes were analyzed by two complementary analyses using the MG-RAST pipeline. First, 16S rRNA gene sequences were retrieved and compared to a database of known 16S rRNA gene sequences (e.g. SSU SILVA rRNA database project). Each read that matched a known sequence was assigned to that organism. In the second analysis putative open reading frames (ORF) were identified and their corresponding protein sequences were searched with BLAST against the M5NR database [18]. The M5NR is an integration of many sequence databases into one single, searchable database. This approach provided us with information for assignments to taxonomic units (e.g. class, families and species) with the caveat a protein sequence could be assigned to more than one closely related organism. Taxonomic assignments were resolved using the lowest common ancestor (LCA) approach [18].

### Functional analysis and reconstruction of metabolic pathways

ORFs were identified and their corresponding protein sequences were annotated (i.e. assigned functions) by comparison to SEED, Pfam, TIGRfam and COG databases [18,19]. Identified proteins were assigned with their respective enzyme commission number (EC). Prior to quantitative characterization, counts were normalized (relative abundance) against the total number of hits in their respective database (e.g. SEED, COG, etc.) using effective sequence counts, a composite measure of sequence number and average genome size (AGS) of the metagenome as described by Beszteri *et al.*[20]. Raes and colleagues [21] defined the AGS as an ecological measure of genome size that also includes multiple plasmid copies, inserted sequences, and associated phages and viruses. Previous studies [20,21] demonstrated that the relative abundance of genes will show differences if the AGS of the community fluctuate across samples. The ChaoI and ACE estimators of COG richness were computed with the software SPADE v2.1 (http://chao.stat.nthu.edu.tw) [22] using the number of individual COGs per unique COG function. The proportion of specific genes in metagenomes also provides a method for comparison between samples. By dividing the AGS to the amount of DNA (in kb) per function-specific gene, one can determine the proportion of genomes in the metagenome that are capable of that function [23]. However, direct comparison of the distribution of different functions (i.e. gene) was not established between the metagenome, since length and copy number of the gene was not incorporated in the formula. To define whether a gene was enriched in the environment we calculated the odds ratio or the relative risk of observing a given group in the sample relative to the comparison dataset [24]. The odds ratios were calculated as follows: (A/B)/(C/D) where A is the number of hits to a given category in the *x* dataset (e.g. TP metagenome), B is the number of hits to all other categories in the *x* metagenome, C is the number of hits to a given category in the *y* dataset (e.g. BP metagenome), and D is the number of hits to all other categories in the *y* dataset. We then used the metagenome profiles to calculate the statistical differences between the two samples based on the Fisher’s exact test with corrected *q*-values (Storey’s FDR multiple test correction approach) using the software package STAMP v1.07 [25]. Such randomization procedures were used to find statistically distinct functional groups in each of the wastewater pipe biofilms. Genes with an odds ratio >1 and *q* < 0.05 were defined as enriched and genes with an odds ratio <1 and *q* < 0.05 as under-represented.

### Taxonomic assignments of metabolic genes

Sequences assigned to the sulfur and nitrogen pathways were identified and retrieved from MG-RAST and RAMMCAP output files (see Metagenomic studies section). Selected genes were taxonomically classified by BLASTX analyses against the NCBI non-redundant protein sequence (nr) database using the CAMERA 2.0 server [26]. Assignment and comparison of taxonomic groups and tree representation of the NCBI taxonomy were performed using the software MEGAN v4.67.1 [27]. The metagenomes were compared at the genus level (when available) using absolute reads counts with default parameters for the lowest common ancestor (LCA) algorithm of min-score of 35, a top-percent value of 10% and min-support of 5.

## Results and discussion

### Metagenome library construction

In this study, we analyzed the microbial communities of biofilms established on the top (TP) and bottom (BP) of a corroded wastewater concrete pipe. The excavated pipe sections were installed 60 years prior to this study and were replaced due to integrity failure resulting from corrosion (i.e. the crown losing a significant portion of original width). A total of 1,004,530 and 976,729 reads averaging 370 and 427 base pairs for the TP and BP metagenomes, respectively, were analyzed in this study (Table 1). We identified and removed artificially replicated reads, which represented a total of 14% and 12% of sequences from the TP and BP metagenomes, respectively. Less than 50% of our reads were annotated as specific genes or functional group by either CAMERA v2 or MG-RAST v3 (Table 1). The relatively low number of annotated genes is common in metagenomic studies [28-30] and is primarily due to the relatively small and biased diversity of genomes sequenced, novel genes yet to be placed in functional groups, and sequencing and processing errors. For diverse and not well-understood systems such as wastewater biofilms, annotation of gene functions can also be limited by the extent of the database of previously sequenced and characterized genes [31]. Nonetheless, high-quality reads with a comparable average genome size were generated in this study, which allowed us to compare the metagenomic data, in terms of what proportion of genomes harbor a particular function [23].

**Table 1:** Characterization of 454 pyrosequenced libraries from the microbial community of biofilms

	**Top pipe (TP)**	**Bottom pipe (BP)**
reads	1 004 530	976 729
avg reads (bp)	370	427
dataset size (10^8^ bp)	3.2	3.7
reads for analysis^§^	862 893	856 080
**CAMERA v2**		
COG hits^†^	370 393	389 807
Pfam hits^†^	338 966	352 466
TIGRfam hits^†^	579 127	607 388
**MG-RAST v3**		
reads matching to a taxa^†^	629 161	641 853
reads matching to a subsystems^†^	425 346	427 295
no. of subsystems (function level)	5 633	6 117
**Annotated proteins (%)** [SEED]		
Bacteria	95.5	94.1
Archaea	0.5	1.3
Virus	0.1	0.1
Eukaryota	0.6	0.3
Unclassified	3.3	4.2
**Comparative metagenome**^‡^		
average genome size [Mb]	3.3	3.3
*ESC* of COG hits	369 671	390 570

^§^Prior to sequence analysis we implemented a dereplication pipeline to identify and remove clusters of artificially replicated sequences [17].

^†^E-value cut-off >1e^-05^.

^‡^Average genome size and effective sequence count (ESC) as calculated by Beszteri *et al.*[20].

### Wastewater biofilms

**Figure 1:**
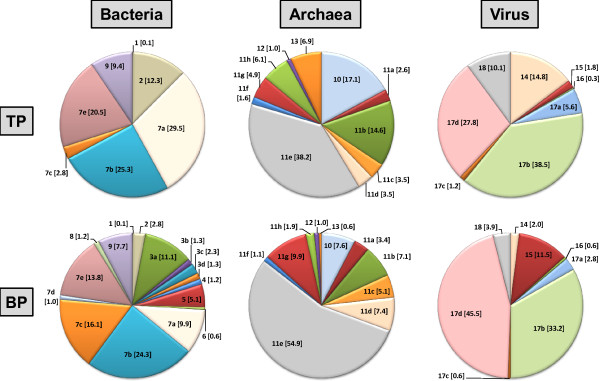
**Distribution of the Bacteria, Archaea and Virus domain as determined by taxonomic identification at class level of annotated proteins.** Numbers in brackets represent percentage of each group from the total number of sequences. **Bacteria domain**: 1. unclassified, 2. *Actinobacteria*, 3a. *Bacteroidia*, 3b. *Cytophagia*, 3c. *Flavobacteria*, 3d. *Sphingobacteria*, 4. *Chlorobia*, 5. *Clostridia*, 6. *Fusobacteria*, 7a. *Alphaproteobacteria*, 7b. *Betaproteobacteria*, 7c. *Deltaproteobacteria*, 7d. *Epsilonproteobacteria*, 7e. *Gammaproteobacteria*, 8. *Synergistia*, and 9. other classes each representing <1%. **Archaea domain**: 10. *Thermoprotei*, 11a. *Archaeoglobi*, 11b. *Halobacteria*, 11c. *Methanobacteria*, 11d. *Methanococci*, 11e. *Methanomicrobia*, 11f. *Methanopyri*, 11g. *Thermococci*, 11h. *Thermoplasmata*, 12. Korarchaeota [phylum] and 13. Thaumarchaeota [phylum]. **Phage (host)**: 14. Actinobacteria, 15. Bacilli, 16. Cyanobacteria, 17a. *Alphaproteobacteria*, 17b. *Betaproteobacteria*, 17c. *Deltaproteobacteria*, 17d. *Gammaproteobacteria* and 18. other classes each representing <1%. Groups (phylum): 3. Bacteroidetes, 7. and 17. Proteobacteria, 10. Crenarchaeota, 11. Euryarchaeota.

The taxonomic classification of 629,161 (TP) and 641,853 (BP) sequence reads was assigned using the SEED database (MG-RAST v3). Based on our results, Bacteria-like sequences dominated both samples (>94% of annotated proteins) (Table 1). Approximately 90% of the total Bacteria diversity was represented by the phyla Actinobacteria, Bacteroidetes, Firmicutes and Proteobacteria (Figure 1). The bacterial community was diverse with representatives of more than 40 classes. Taxonomic annotation of the functional genes profiles (i.e. annotated proteins) displayed a similar pattern of diversity to taxonomic analysis based on 16S rRNA genes identified from the metagenome libraries ( Additional file 1, Figure S2).

Some annotated proteins were associated with archaeal genes, and to a lesser extent to viral and eukaryotic genes (Table 1, Figure 1). Specifically, a total of 2,837 (TP) and 8,237 (BP) Archaea-related functions were identified using the SEED database. The majority of the annotated sequences in both samples were related to proteins affiliated with archaea members of the class *Methanomicrobia*. Although, phages are extremely abundant and diverse in natural systems, we were able to identify only a low number of sequences (696), perhaps due to the loss of viruses during the sample concentration or DNA extraction steps [32]. Nonetheless, the results indicated that the community composition and structure of viruses parallels the distribution of Bacterial representatives [33]. Specifically, phages associated to the classes Actinobacteria, Alphaproteobacteria, Betaproteobacteria, Gammaproteobacteria and Deltaproteobacteria were found to be the dominant phage sequences in our metagenomes (Figure 1). Phages can potentially be used as biocontrol agents to specifically control some of the bacteria implicated in corrosion. Future studies should focus on the use of viral concentration methods to further study the occurrence of phage sequences that could be use as targets to monitor biocorrosion bacteria in wastewater concrete pipes.

### Comparative microbial community analysis

In previous studies, biofilms were analyzed from the surface of primary settling tanks from a domestic wastewater treatment plant [7,8] and from coupons placed in a collection system manhole [9], while our study focused on biofilms from top and bottom of a corroded pipe. In spite of the differences in sample matrix, some trends in the bacterial distribution between concrete wastewater biofilms were observed ( Additional file 1, Figure S3). For example, the bottom of the pipe (BP) is characterized by direct contact and long residence time with wastewater, which maintains an ideal anaerobic environment for SRB. In fact, obligate anaerobes of the class *Deltaproteobacteria* (16%) were the dominant cluster in BP biofilm (Figure 1). The BP harbored anaerobic bacteria normally found in the human gut such as members of the *Bacteroidia* (11%) and *Clostridia* (5.1%) classes (Figure 1 and Additional file 1, Figure S2). This was also supported by data from 16S rRNA gene clone libraries (Additional file 1, Figure S 4). We also retrieved sequences from the gut-related archaeal species *Methanobrevibacter smithii* in the BP metagenome [34]. These findings are not surprising, as human fecal bacteria has also been noted in concrete biofilms in previous studies [7-9].

Sections of wastewater pipes exhibit conditions that are favorable for the establishment of oxic zones, e.g., at the top of the pipe (TP). In fact, the dominant TP biofilm members were associated with aerobic and facultative anaerobic bacteria (e.g. *ThiobacillusAcidiphiliumXanthomonasBradyrhizobium*). The biofilms did not contain a significant presence of photosynthetic organisms (e.g. *Cyanobacteria*), which dominated biofilms in concrete corroded city-surface structures [10]. The latter is supported by the low number of genes assigned to the photosynthesis subsystems in our metagenome libraries ( Additional file 1, Figure S1).

Taxonomic analysis based on annotated proteins show two distinct archaeal communities (Figure 1). The BP biofilm was dominated by the classes *Methanomicrobia* (55%), *Thermococcus* (10%) and *Thermoprotei* (8%). The classes *Methanomicrobia* (38%) and *Thermoprotei* (17%) were also abundant in the TP site although *Halobacteria* (15%) and Thaumarchaeota (7%) were also abundant. Members of the Thaumarchaeota phylum are chemolithoautotrophic ammonia-oxidizers, which suggest that they may be playing a role in the nitrogen cycle in wastewater concrete biofilms [35]. *Halobacteriales* have been previously reported in wastewater sludge and may suggest the presence of alkaline hypersaline microenvironments in wastewater concrete biofilms [36]. The anaerobic niches in the wastewater pipe provide conditions for methanogenesis as suggested by the annotated sequences associated with genera such as *MethanospirillumMethanobrevibacterMethanosphaeraMethanosaetaMethanosarcina*, and *Methanococcoides*[37]. However, the more favourable anaerobic conditions at the bottom of the pipe provide better conditions for this process. Indeed, there are a higher percentage of annotated sequences related to methanogenesis in the BP (69%) than in TP metagenomes (47%). Conversely, more methanotrophic and methylotrophic bacteria proteins were present in the TP (3.7%) than in BP biofilm (1.8%). Specifically, many of the sequences were related to proteins affiliated with *MethylibiumMethylobacillusMethylobacteriumMethylocellaMethylococcus*, and *Methylacidiphilum*. The dominant annotated methane-oxidizing bacteria in the TP biofilm were affiliated with *Methylocella silvestris*, a moderately acidophilic (pH values between 4.5 and 7) and mesophilic species [38]. In general, our analysis identified microorganisms associated with one-carbon compound pathways (e.g. methanogenesis, methanotrophs and methylotrophs), although the importance of these metabolic processes in wastewater pipes remains unknown.

### The role of biofilms in MICC

Anaerobic conditions in wastewater collection systems support sulfate reducing bacteria (SRB) that convert sulfate and organic sulfides to H_2_S, which volatilizes to the sewer atmosphere and redissolves on the top of the pipe. The microbial community at the top oxidizes the sulfide to corrosive H_2_SO_4_[39]. Consistent with this observation, analysis of 16S rRNA gene clone libraries showed that the community structures differ, with a dominant presence in the BP of sulfate reducing bacteria (SRB) affiliated to *Deltaproteobacteria*. Specifically, there were 24 phylotypes represented by the genera *DesulfobacterDesulfobacteriumDesulfobulbusDesulfomicrobiumDesulforegula* and *Desulfovibrio* (Additional file 1, Figure S 5). The predominant SRB phylotype (5.4%) in the clone libraries is closely related to *Desulfobacter postgatei*, a strict anaerobic chemoorganotroph that completely oxidizes acetate to CO_2_ and reduces sulfur compounds (e.g. sulfate, sulfite, or thiosulfate) to H_2_S [40]. In the TP sample, most SOB phylotypes (i.e., 39 of 45) are affiliated to the genus *Thiobacillus* (Betaproteobacteria) ( Additional file 1, Figure S6), further supporting the importance of this group in concrete corrosion [41]. During the concrete corrosion process it has been shown that *Thiobacillus thioparusT*. *novellusT*. *neapolitanus*, and *T*. *intermedius* are involved in the initial and intermediate stages of colonization, while *T*. *thiooxidans* dominate in the final stage when the pH reaches values <3 [3]. In our study the majority of the *Thiobacillus*-like sequences were closely related to uncultured sulfur-oxidizing bacteria clones. Interestingly, two of the dominant clones in our libraries were identified as neutrophilic *T*. *thioparus* and *T*. *plumbophilus* (>98.5% sequence identity) (Additional file 1, Figure S 6). *T*. *thioparus* oxidizes sulfur and thiosulfate, reducing the medium between pH 3.5 and 5 [3]. *T*. *plumbophilus* grows by oxidation of H_2_S and H_2_ at pH 4 and 6.5 [42]. There were also sequences with a high sequence homology (>99%) to representatives of the *Thiomonas intermedia* and *Acidiphilium acidophilum*, members of the *Beta*- and *Alphaproteobacteria* class, respectively*. T*. *intermedia* is an obligate aerobe and facultative chemolithoautotroph that produces sulfuric acid at an optimum pH between 5 and 7 [43]. *Thiomonas* species are unable to denitrify or oxidize ferrous iron. In contrast, *A*. *acidophilum* is able to grow autotrophically or mixotrophically using sulfur or reduced inorganic sulfur compounds, as well as heterotrophically using various organic compounds and is capable of reducing iron [44].

Wastewater concrete corrosion involves the interaction of multiple groups and the establishment of these groups are driven by factors, such as the pH of the concrete, and the temporal dynamics of sulfur compounds [41]. The data from different studies conducted thus far suggest that the composition of species involved in concrete corrosion may vary within different wastewater systems. For instance, our study did not find any hyper-acidophilic SOB sequences (e.g. *T. thiooxidans, Acidithiobacillus thiooxidans*) which had been previously detected in various MICC studies [39]. Okabe and colleagues [8] did not find *T*. *thioparus*, although *A*. *acidophilum* and *T*. *plumbophilus* were present at several stages of the MICC process. Altogether, molecular surveys strongly indicate that the dynamics of multiple microbial groups need to be studied in order to better develop condition assessment tools to monitor the performance of biocorrosion control measures.

### Comparative metagenome analysis

Analysis of annotated COG (ChaoI and *S*_ACE_: ≈3932) also showed that the wastewater biofilm samples are highly diverse. The level of COG diversity is similar to that described for whale fall (3,332), soil (3,394), and Sargasso Sea samples (3,714), but higher than that described for acid mine drainage (1,824) and human distal gut (2,556) [24,45]. Statistical tests based on COG categories or SEED subsystems found no significant difference in community richness between the BP and TP samples (*t*-test, *p* = 0.156). The majority of the assigned genes in both metagenomes were identified as part of the SEED database Carbohydrate subsystem (Additional file 1, Figure S 1) with sequences linked to CO_2_ fixation, Central Carbohydrate and Fermentation subsystems. In both biofilms the single most abundant component of the Carbohydrate subsystem was the TCA Cycle followed by the significant presence of common functions involved in Glycolysis and Gluconeogenesis, Photorespiration (oxidative C2 cycle), Pentose phosphate pathway, Entner-Doudoroff Pathway, Trehalose Biosynthesis and CO_2_ uptake. There were distinctive differences between the metagenomes in the Carbohydrate subsystem (Fisher’s exact test, *q* < 0.05). A significant number of sequences in the TP were associated with CO_2_ fixation and included CO_2_ uptake (carboxysome) and photorespiration (oxidative C2 cycle). Carboxysomes are microcompartments that enhance the fixation of CO_2_ by RuBisCO and are present in several chemoautotrophic bacteria, including sulfur bacteria, such as *Thiobacillus denitrificansT. intermedia*, and *A. ferrooxidans*[46]. Most of the BP sequences shared homologies to known genes involved in pyruvate:ferredoxin oxidoreductase, lactose utilization, β-glucoside metabolism, mixed acid fermentation, organic acids utilization (e.g. lactate) and sugar alcohols utilization (e.g. ethanolamine and propanediol). Based on the functional metabolic profile, the data suggest that the community present in the BP is predominantly composed of anaerobic or facultative aerobic bacteria with a wide variety of metabolic functions (Additional file 1, Figure S 1). A relative high number of sequences were associated with cell maintenance and structural functions such as cell division, cell wall and synthesis of DNA, RNA and proteins. Consistent with other environments, individual biochemical pathways (e.g. Nitrogen, Sulfur, Iron, Phosphorous and Potassium) comprised less than 1% of the functional genes profile [47,48]. Although functional similarities were observed, there were also relevant differences between the two biofilm samples. Most of the differences were attributed to the enrichment of specific gene families within metabolic pathways, some of which may indicate functional niches corresponding to varying microenvironments in the sewer pipes.

### Sulfur metabolism

**Figure 2:**
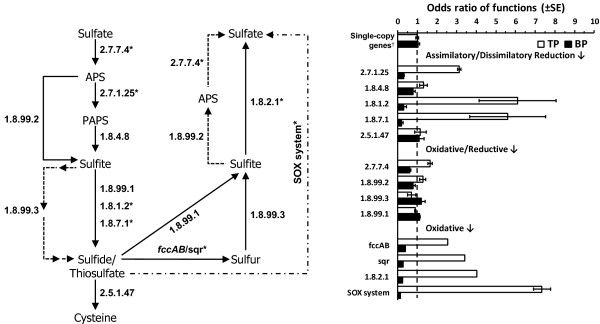
**Enrichment of enzymes in the sulfur metabolic pathway.** Diagram with the enzyme classification (identified by their Enzyme Commission number; EC number) for each step in the sulfur pathway. Asterik (*) indicate components that are significantly different between the two samples (*q* < 0.05) based on the Fisher’s exact test using corrected *q*-values (Storey’s FDR multiple test correction approach) (Table 2). Bar chart shows the odds ratio values for each function. An odds ratio of 1 indicates that the community DNA has the same proportion of hits to a given category as the comparison data set [24]. Housekeeping genes: *gyrAgyrBrecArpoA* and *rpoB*. Error bars represent the standard error of the mean.

Analysis of metagenome libraries identified key genes implicated in the sulfur pathway (Figure 2). These functions were found to be abundant in the metagenomes, although we observed differences in the enrichment of specific gene families within the sulfur pathway. For example, in both metagenomes enzymes of three pathways involved in sulfur oxidation were detected: the Adenosine-5’-Phosphosulfate (EC 2.7.7.4, EC 1.8.99.2), the Sulfite:Cytochrome C oxidoreductase (EC 1.8.2.1) and the Sox enzyme complex (Figure 2). However, we found a relatively low odds ratio for the first pathway (<1.5), while the enzymes of the Sox complex that convert thiosulfate to sulfate were more statistically abundant and enriched (odds ratio >9) in the TP biofilm (Fisher’s exact test, *q* < 0.05) (Table 2, Figure 2). Approximately 66% of the genomes in TP metagenome contained the *soxB* gene, a key gene of the periplasmic Sox enzyme complex [49] (Table 2). The widespread distribution of the Sox-complex among various phylogenetic groups of SOB was confirmed [50], specifically *soxB*-sequences affiliated with *T*. *intermediaT*. *denitrificansT*. *thioparusAcidiphilium cryptum*, and species of *Burkholderia* among others ( Additional file 1, Figure S7). The relative similar level of enrichment of the Adenosine-5’-Phosphosulfate pathway may be explained by the fact that key enzymes can be found in species of SRB and SOB, in which the latter can operate in the reverse direction [51,52]. In addition, the composition of species carrying the *dsrB* gene (sulfite reductase; EC 1.8.99.1) is noteworthy (Fisher’s exact test, *q* < 0.05) (Figure 2 and Table 2). Retrieved *dsrB*-sequences for the TP biofilm show 80% of genes were closely related to *T. denitrificans* (SOB), while 78% in the BP were represented by SRB: *Desulfobacter postgateiDesulfomicrobium baculatum*, and species of *Desulfovibrio* among others ( Additional file 1, Figure S7).

**Table 2:** Estimation (%) and enrichment of Sulfur and Nitrogen biochemical functional genes in wastewater genomes

**Subsystem**	**Gene**	***n***	**% of genomes with gene**^†^		*q*-value*	**Odds ratio**	
			**TP**	**BP**		**TP/BP**	**BP/TP**
**Single-copy genes**^‡^		5	100	100	ns	1.0	1.0
**Sulfur metabolism**							
Sulfate adenylyltransferase (ATP)	*cysN*	1	54	33	0.000	1.6	0.6
Adenylyl-sulfate kinase	*aspK*	1	52	15	0.000	3.2	0.3
Phosphoadenylyl-sulfate reductase	*cysH*	1	26	22	ns	1.1	0.9
Adenylyl-sulfate reductase	*aprA*	1	15	10	ns	1.4	0.7
3'(2'),5'-bisphosphate nucleotidase	*cysQ*	1	67	40	0.000	1.6	0.6
Hydrogensulfite reductase	*dsrA*	1	13	15	ns	0.8	1.3
Sulfite reductase (NADPH)	*cysJ*	1	28	4	0.000	7.6	0.1
Sulfite reductase (DSR)	*dsrB*	1	13	14	ns	1.0	1.0
Sulfite reductase (ferredoxin)	*sir*	1	22	6	0.000	3.7	0.3
Cysteine synthase	*cysK*	1	>100	>100	ns	1.0	1.0
Thiosulfate oxidise	*soxB*	1	66	7	0.000	9.1	0.1
**Nitrogen metabolism**							
Ammonia monooxygenase	*amoA*	1	8	29	0.000	0.3	3.6
Nitrate reductase	*napA*	1	2	13	0.000	0.1	8.0
Nitrate reductase	*narG*	1	17	28	0.000	0.6	1.7
Nitrate reductase	*nasA*	1	68	34	0.000	2.0	0.5
Nitric oxide reductase	*norB*	1	2	23	0.001	0.1	9.4
Nitric oxide reductase	q*nor*	1	22	23	ns	1.0	1.0
Nitrite reductase	*nirK*	1	17	3	0.000	5.2	0.2
Nitrite reductase	*nirS*	1	2	30	0.000	0.1	16.4
Nitrous oxide reductase	*nosZ*	1	10	35	0.030	0.3	3.6
Nitrite reductase	*nirB*	1	64	44	0.000	1.4	0.7
Nitrite reductase	*nirA*	1	7	1	0.018	5.6	0.2
Nitrite reductase	*nrfA*	1	1	45	0.000	0.0	58.4
Nitrogenase (molybdenum-iron)	*nifD*	1	1	23	0.000	0.0	24.6
Nitrogenase (iron)	*nifH*	1	15	23	0.006	0.6	1.6

*Indicate components that are significantly different between the two samples (*q* < 0.05) based on the Fisher’s exact test using corrected *q*-values (Storey’s FDR multiple test correction approach).

^‡^Housekeeping genes: *gyrA*, *gyrB*, *recA*, *rpoA* and *rpoB*.

^†^Direct comparison between the frequency of different functional genes, either within or between metagenomes, was not established since length and copy number of the gene was not incorporated in the formula.

*TP*: top pipe.

*BP*: bottom pipe.

*NS*: not significant.

*ND*: not determine.

The wide range of annotated functions associated in several sulfur pathways may be indicative of the availability of several electron donors at wastewater pipes undergoing corrosion. While the role of some bacterial groups might be predicted based on previous studies, our study suggests that additional bacterial groups might be playing important roles within wastewater concrete corrosion processes. This is the case for SRB as they are a phylogenetically diverse group that cannot be monitored using a single 16S rRNA gene assay ( Additional file 1, Figure S7). Our approach provides a sequence-based framework that can be used to monitor relevant microbial populations via function-specific assays. These assays can be used to measure the expression of key genes involved in corrosion processes, and hence be used to provide a condition assessment tool prior to corrosion processes that are irreversible.

### Nitrogen metabolism

**Figure 3:**
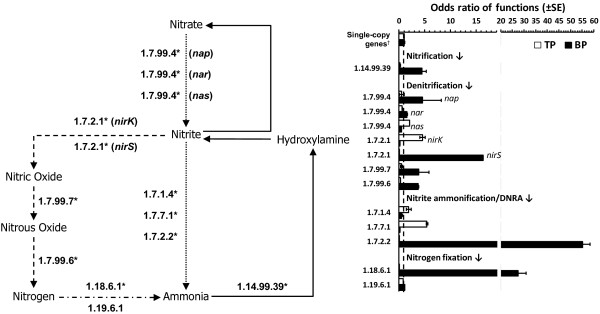
**Enrichment of enzymes in the nitrogen metabolic pathway.** Diagram with the enzyme classification (identified by their Enzyme Commission number; EC number) for each step in the nitrogen pathway. Asterik (*) indicate components that are significantly different between the two samples (*q* < 0.05) based on the Fisher’s exact test using corrected *q*-values (Storey’s FDR multiple test correction approach) (Table 2). Bar chart shows the odds ratio values for each function. An odds ratio of 1 indicates that the community DNA has the same proportion of hits to a given category as the comparison data set [24]. Housekeeping genes: *gyrAgyrBrecArpoA* and *rpoB*. Error bars represent the standard error of the mean.

In spite of the importance of the nitrogen cycle in a wide range of habitats, the functional capabilities and distribution of their enzymes in wastewater systems, such as concrete biofilms, have not been fully explored. We identified key genes for nitrification, denitrification, nitrogen fixation and nitrate ammonification, including ammonia monooxygenase (*amoA*), nitrate reductase (*narGnapAnasA*), nitrite reductase (*nirKnirS*), nitric oxide reductase (nor), nitrous oxide reductase (*nosZ*), nitrogenase (*nifHnifD*) and assimilatory nitrite reductase (*nrfAnirAnirB*) in both metagenomes (Figure 3). Differences in the distribution and taxonomic assignment of key genes involved in the nitrogen cycle were observed in our analysis (Table 2 and Additional file 1, Figure S8). Specifically, *amoAnarGnapAnirS* and *nrfA* were highly enriched in the BP sample, while there was a higher distribution of the *nasAnirK* and *nirB* in the TP (Fisher’s exact test, *q* < 0.05). The majority of the sequences in the BP sample were annotated to species of *AcidovoraxThauera* and Deltaproteobacteria (i.e. SRB), while most of the genes in the TP were associated with members of the *T*. *intermediaT*. *denitrificans*, and species of *Burkholderia* among others (Additional file 1, Figure S 8). Differences in the distribution and functional capability may be associated with the availability of oxygen and concentration of N compounds at each environment. Respiratory nitrate reductase (*narG*) reduces nitrate to nitrite predominantly during anaerobic growth, while the *nasA* assimilate nitrate during aerobic growth [53]. Furthermore, the enrichment of *nirSnor*, and *nosZ* suggest that the majority of the nitrite in the BP biofilm is reduced preferentially through the denitrification pathway (Figure 3). The *nrfA* enzyme is highly enriched at the BP biofilm (Fisher’s exact test, *q* < 0.05) (Figure 3 and Table 2), supporting the observation that the *nrfA* enzyme is expressed when nitrate (or nitrite) is limiting in the environment [54]. On the other hand, we observed an enrichment of the *nirB* at the TP biofilm (Fisher’s exact test, *q* < 0.05) (Figure 3 and Table 2), which is expressed only when nitrate or nitrite is in excess in the environment [54]. The enrichment of nitrification genes in the BP may be explained by the fact that domestic wastewater carry a substantial concentration of nitrogen compounds (20 to 70 mg/L), consisting of 60-70% NH_3_‒N and 30-40% organic N [55]. In fact, the gene encoding for ammonia monooxygenase (*amoA*), a key enzyme for ammonia oxidation was highly enriched in the BP metagenome (Fisher’s exact test, *q* < 0.05) (Table 2). The metagenome data suggest that habitat prevailing conditions can select for bacterial populations with functionally equivalent yet ecologically nonredundant genes [56]. Specifically, we noted *nirK* is enriched in the TP while the *nirS* (nitrite reductase) is more prevalent in the BP biofilm (Fisher’s exact test, *q* < 0.05).

### Functional diversity

We detected the presence of several types of adaptive responses to various heavy metal ions with the majority of the heavy metal-related functions enriched in the TP biofilms where the acid conditions are prevalent (Table 3). The majority of heavy metals become more soluble and mobile under low pH conditions [57]. It also appears that TP and BP biofilms are dominated by different types of uptake systems to control the intracellular concentration of heavy metal ions: (1) a fast, unspecific and constitutively expressed system and (2) an ATP hydrolysis-dependent slower yet highly specific system [58]. For example, the stand-alone *arsB* chemiosmotic transport protein (i.e. anion channel) is enriched in the TP biofilm (Fisher’s exact test, *q* < 0.05), while the BP biofilm is rich in *arsA* enzymes (EC 3.6.3.16) (Fisher’s exact test, *q* < 0.05), which transform the *arsB* into an *arsAB* ATPase complex [59]. The presence of heavy metal compounds provide the opportunity for selected individuals to oxidize these substrates and generate energy, as is the case of the presence of *Thiomonas* spp. with *aoxB* arsenite oxidase genes (EC 1.20.98.1) [60].

**Table 3:** Estimation (%) and enrichment of motility, stress, antibiotics and toxic resistance genes in wastewater genomes

**Subsystem**	**Gene**	***n***	**% of genomes with gene**^†^		*q*-value*	**Odds ratio**	
			**TP**	**BP**		**TP/BP**	**BP/TP**
**Single-copy genes**^**‡**^		5	100	100	ns	1.0	1.0
**Heavy metal resistance**							
Arsenate reductase (glutaredoxin)	*arsC*	1	50	17	0.000	2.8	0.4
Arsenic efflux pump protein	*arsB*	1	24	10	0.000	2.4	0.4
Arsenic resistance protein	*arsH*	1	37	5	0.000	7.4	0.1
Arsenical pump-driving (ATPase)	*arsA*	1	15	28	0.000	0.5	1.9
Arsenite oxidase	*aoxB*	1	10	8	ns	1.3	0.8
Cadmium-transporting (ATPase)	*cadA*	1	3	14	0.000	0.2	4.5
Chromate transport protein	*chrA*	1	40	50	0.034	0.8	1.3
Copper-translocating P-type (ATPase)	*copA*	1	>100	>100	ns	1.1	0.9
CZC resistance protein	*czcD*	1	>100	75	0.006	1.6	0.6
Mercuric reductase	*merA*	1	80	33	0.000	2.4	0.4
**Antibiotics & toxicity resistance**							
Beta-lactamase	*ampC*	1	>100	>100	0.000	1.8	0.6
Beta-lactamase (MRSA)	*mecA*	1	0	0	nd	0	0
Dihydrofolate reductase	*folA*	1	80	47	0.034	1.6	0.6
Pterin binding enzyme	*sul*	1	83	66	0.003	1.3	0.8
Multidrug efflux system protein	*acrB*	1	>100	>100	0.000	1.4	0.7
Dioxygenase (Bleomycin resistance)	*bleO*	1	>100	>100	0.000	2.3	0.4
Aminoglycoside-3’-adenylyltransferase	*aadA*	1	40	>100	0.000	0.3	3.2
Antiholin-like protein (murein hydrolase)	*lrgA*	1	4	37	0.000	0.1	9.6
Antiholin-like protein (murein hydrolase)	*lrgB*	1	17	39	0.001	0.4	2.5
Streptomycin adenylyltransferase	*ant1*	1	0	3	0.031	0.0	nd
Drug resistance transporter	*cflA*	1	61	37	0.000	1.6	0.6
MFS transporter (DHA2)	*emrB*	1	>100	57	0.000	3.6	0.3
D-alanine--D-alanine ligase	*vanA*	1	76	81	ns	0.9	1.1
Multi antimicrobial extrusion protein	*norM*	1	6	40	0.000	0.2	6.6
Multidrug efflux transporter	*mexF*	1	16	6	0.043	2.7	0.4
RND efflux system (transporter)	*cmeB*	1	53	>100	0.000	0.5	2.1
RND efflux system (membrane protein)	*cmeA*	1	18	46	0.005	0.4	2.5
RND efflux system (lipoprotein)	*cmeC*	1	19	60	0.020	0.3	3.1
**Protein secretion systems**							
Type I	--	1	nd	nd	0.000	1.5	0.7
Type III	--	10	nd	nd	0.001	0.8	1.8
Type IV	--	5	nd	nd	0.000	3.1	1.4
Type V	--	3	nd	nd	0.001	1.7	0.6
Type VI	--	10	nd	nd	0.000	2.8	0.7
**Motility & Chemotaxis systems**							
motility/chemotaxis	--	74	nd	nd	0.000	0.7	2.7
**Stress systems**							
stress response	--	276	nd	nd	0.000	2.2	1.8

*Indicate components that are significantly different between the two samples (*q* < 0.05) based on the Fisher’s exact test using corrected *q*-values (Storey’s FDR multiple test correction approach).

^‡^Housekeeping genes: *gyrA*, *gyrB*, *recA*, *rpoA* and *rpoB*.

^†^Direct comparison between the frequency of different functional genes, either within or between metagenomes, was not established since length and copy number of the gene was not incorporated in the formula.

*TP*: top pipe.

*BP*: bottom pipe.

*NS*: not significant.

*ND*: not determine.

A high number of genes associated with motility, stress response, antibiotic resistance, and virulence (e.g. efflux pump) were also identified in this study (Table 3). Motility and chemotaxis related functions seem to be important properties for submerged environments, such as the BP site, enabling bacteria to rapidly colonize surfaces through biofilm formation [61] and to respond to changes in environmental conditions characteristic of wastewater habitats [62]. In extreme and rapidly changing habitats, such as corroded concrete structures, microorganisms must respond with appropriate gene expression and protein activity [63]. We detected the enrichment of stress response components at the TP, which is characterized by the low pH of the surface and temporal changes in heavy metal ions due to corrosion (Table 3). Both biofilms have a high distribution of genes related to antibiotic resistance with a significant percentage of the genes incorporated in their genomes (Table 3). Furthermore, the wastewater biofilms contained an abundance of virulence-associated protein secretion systems, representing a reservoir for virulence genes. This may represent a conservative estimate of the number of potential virulence factors, since we only screened for a subset of genes homologous to type I, IV, V and VI secretion systems [64]. The significant number of resistance and virulence genes in their genomes and distribution based on odds-ratio (i.e. enrichment) analysis is consistent with the idea that sewage systems harbor favorable conditions for the establishment and propagation of antibiotic resistant bacteria [65].

Metagenomic data generated in this study enabled us to detect, identify and reconstruct metabolic pathways involved in MICC. The information generated from these sequencing libraries will help us better understand the genetic network and microbial members involved in wastewater biofilms. This information is also relevant to track microbial populations associated with concrete biofilms and to evaluate molecular assays used to detect key functional genes. In a recent study, Santo Domingo and colleagues [11] failed to detect the presence of ammonia oxidizing bacteria (AOB) on wastewater concrete biofilms using *amoA*-based PCR assays. These bacteria are expected to be associated with wastewater systems. In this study we were able to detect the presence of putative membrane-associated ammonia monooxygenase in the BP biofilm. The metagenomic sequences were highly homologous to sequences from heterotrophic representatives of the species *Acidovorax delafieldiiThauera* sp MZ1T and species of Rhizobiales (Additional file 1, Figure S 8). Heterotrophic ammonia oxidizing bacteria are commonly found in wastewater systems [66]. Ammonia oxidation by heterotrophic bacteria usually does not involve the generation of energy and is probably used as a sink for excess reducing power generated by oxidative metabolism [67]. Thus, the lack of previous detection of *amoA* genes by Santo Domingo *et al.*[11] can be explained by the fact that the assay cannot detect the *amoA* in heterotrophic ammonia oxidizing bacteria as they were designed to amplify representatives of the autotrophic ammonia monooxygenase, for example, *Nitrosomonas* species [68]. On the other hand, this study confirmed the validity of the *soxB* PCR-based assay to detect the presence of thiosulfate-oxidizing Sox enzyme complex in wastewater concrete [11]. A high percentage (>90%) of our metagenome sequences belong to species that contain the region for the Sox primers designed by Petri and colleagues [69], suggesting that they can be used to ascertain the presence of SOB in this environment.

In wastewater collection systems the sulfur and nitrogen pathways play an important role in MICC, and the populations engaged in these pathways are part of a complex and highly diverse microbial community [39]. The reconstruction of the sulfur metabolism network showed several pathways used to oxidize the end products of sulfate reduction leading to the production of H_2_SO_4_, e.g. Sox complex, sulfide quinone oxidoreductase (*sqr*) and the flavocytochrome *c* (*fccAB*) in the corroded section of the pipe (Figure 2). We detected similar levels of enrichment in both biofilms of the *dsrB* enzyme (Table 3). On the basis of these observations, and to better understand and control MICC, future investigations must consider the ability of these communities to: (1) utilize different sulfur compounds, e.g. thiosulfate (Sox complex) or sulfide (*sqrfccAB*), (2) adapt to temporal variation in the concentrations of sulfide, e.g. low sulfide (*sqr*) and high sulfide (*fccAB*), and (3) reverse the action of their enzymes, e.g. *dsrB* involves both the oxidative and the reductive mode of the dissimilatory sulfur metabolism. Sequences obtained in this study provide the molecular framework to detect the populations carrying relevant functions in future monitoring studies ( Additional file 1, Figures S7 and S 8).

Recently safe and cost-effective approaches to inhibit or prevent corrosion have included influencing the microbial population without the application of biocides by (1) supporting the establishment of competitive biofilms and (2) removing or adding electron acceptors such as nitrate [5,70]. The addition of nitrate can stimulate the growth of competing bacterial populations (e.g. nitrate-reducing bacteria), which can effectively displace the SRB [71]. The success of these approaches must include a detailed analysis of the established bacterial populations and functional capabilities of the microbial community in that particular system. In fact, our data provide evidence of the effect of habitat selective factors on microorganisms and consequently their functional capabilities. For example, the diversity of the denitrification genes *nirK* and *nirS* increased in habitats with relatively moderate and low levels of nitrate/nitrite, respectively [72]. Other corrosion control approaches include commercially available coating techniques, for which limited data is available on their performance. The data from this study identified the potential bacterial groups and specific gene sequences that remediation approaches need to target to prevent microbial colonization of key concrete corrosion-associated microbiota.

## Conclusions

In the present work, we analyzed wastewater concrete metagenomic and phylogenetic sequences in an effort to better understand the composition and function potential of concrete biofilms. The analyses unveiled novel insights on the molecular ecology and genetic function potential of concrete biofilms. These communities are highly diverse and harbor complex genetic networks, mostly composed of bacteria, although archaeal and viral (e.g., phages) sequences were identified as well. In particular, we provided insights on the bacterial populations associated with the sulfur and nitrogen cycle, which may be directly or indirectly implicated in concrete corrosion. By identifying gene sequences associated with them, their potential role in the corrosion of concrete can be further studied using multiple genetic assays. The development of comprehensive databases such as the one generated in this study as well as for microbial communities in wastewater systems with a wide range of corrosion conditions will be useful in the development of tools in diagnosing and preventing MICC. Although the emphasis of this study was on corrosion processes, we also identified the presence of bacterial virulence factors and antibiotic resistance genes, suggesting that these systems are reservoirs of microbial populations of public health relevance.

## Authors’ contributions

VGA participated in bioinformatic and statistical analyses. RPR and JSD carried out sample collection and sample processing. RPR and JSD participated in design and coordination of the study. JSD conceived of the study. All authors helped to draft and revise the manuscript. All authors read and approved the final manuscript.

## Supplementary Material

Additional file 1 **Figure S1. Distribution (%) of sequences identified to particular subsystems (SEED) in metagenomes of wastewater biofilms.Figure S2.** Distribution of bacterial classes on concrete wastewater pipes as determined by taxonomic identification of 16S rRNA genes recovered from metagenome libraries. Numbers in brackets represent percentage of each group from the total number of sequences. Legend: 1. unclassified Bacteria domain, 2. *Actinobacteria*, 3a. *Bacteroidia*, 3b. *Flavobacteria*, 3c. *Sphingobacteria*, 4. *Chloroflexi*, 5a. *Bacilli*, 5b. *Clostridia*, 6. Fusobacteria, 7a. *Alphaproteobacteria*, 7b. *Betaproteobacteria*, 7c. *Deltaproteobacteria*, 7d. *Epsilonproteobacteria*, 7e. *Gammaproteobacteria*, 8. *Synergistia* and 9. other classes each representing <1%. Groups (phylum): 3. Bacteroidetes, 5. Firmicutes, 7. Proteobacteria . **Figure S3.** UPGMA cluster analysis of Bray-Curtis similarity coefficients for biofilms in wastewater systems. Sample types were classified by their taxonomic dominant group within the sulfur biogeochemical cycle: sulfur-reducing bacteria (SRB) and sulfur/sulfide-oxidizing bacteria (SOB). Location of biofilm: bottom (a), middle (b), top (c) and outdoor (d). **Figure S4.** Phylogenetic affiliation of phylotypes identified as *Bacteroidetes* from each biofilm: top pipe (TP, gray) and bottom pipe (BP, black). Clones were identified by genus or order (*) and percentage of each representative sequence in their respective libraries is provided in the brackets. The tree was inferred using maximum likelihood analysis of aligned 16S rRNA gene sequences with bootstrap values from 100 replicates. Box indicates the two most dominant phylotypes. **Figure S5.** Phylogenetic affiliation of *Deltaproteobacteria* phylotypes identified as sulfate-reducing bacteria (SRB) from each biofilm: top pipe (TP, gray) and bottom pipe (BP, black). Clones were identified by genus or family (*) and percentage of each representative sequence in their respective libraries is provided in the brackets. The tree was inferred using maximum likelihood analysis of aligned 16S rRNA gene sequences with bootstrap values from 100 replicates. Box indicates dominant phylotype**. Figure S6.** Phylogenetic affiliation of the top 20 most abundant Proteobacteria phylotypes identified as sulfur/sulfide-oxidizing bacteria (SOB) from each biofilm: top pipe (TP, gray) and bottom pipe (BP, black). Clones were identified by genus (*family) and percentage of each representative sequence in their respective libraries is provided in the brackets. The tree was inferred using maximum likelihood analysis of aligned 16S rRNA gene sequences with bootstrap values from 100 replicates. Box indicates dominant phylotype **Figure S7.** Relative abundance of taxonomic groups based on MEGAN analysis of protein families associated with the sulfur pathway. Each circle is scaled logarithmically to represent the number of reads that were assigned to each taxonomic group. Wastewater biofilms: top pipe (TP, white) and bottom pipe (BP, black). EC = Enzyme Commission number. **Figure S8.** Relative abundance of taxonomic groups based on MEGAN analysis of protein families associated with the nitrogen pathway. Each circle is scaled logarithmically to represent the number of reads that were assigned to each taxonomic group. Wastewater biofilms: top pipe (TP, white) and bottom pipe (BP, black). EC = Enzyme Commission number. Click here for file
