# Supplementary material for: Metagenome analyses of corroded concrete wastewater pipe biofilms reveal a complex microbial system
Source: BMC Microbiol. 2012 Jun 22;12:122. doi: 10.1186/1471-2180-12-122 (PMC3409016; doi:10.1186/1471-2180-12-122)
Supplement: Additional file 1 — Figure S1. Distribution (%) of sequences identified to particular subsystems (SEED) in metagenomes of wastewater biofilms.Figure S2. Distribution of bacterial classes on concrete wastewater pipes as determined by taxonomic identification of 16S rRNA genes recovered from metagenome libraries. Numbers in brackets represent percentage of each group from the total number of sequences. Legend: 1. unclassified Bacteria domain, 2. Actinobacteria, 3a. Bacteroidia, 3b. Flavobacteria, 3c. Sphingobacteria, 4. Chloroflexi, 5a. Bacilli, 5b. Clostridia, 6. Fusobacteria, 7a. Alphaproteobacteria, 7b. Betaproteobacteria, 7c. Deltaproteobacteria, 7d. Epsilonproteobacteria, 7e. Gammaproteobacteria, 8. Synergistia and 9. other classes each representing <1%. Groups (phylum): 3. Bacteroidetes, 5. Firmicutes, 7. Proteobacteria . Figure S3. UPGMA cluster analysis of Bray-Curtis similarity coefficients for biofilms in wastewater systems. Sample types were classified by their taxonomic dominant group within the sulfur biogeochemical cycle: sulfur-reducing bacteria (SRB) and sulfur/sulfide-oxidizing bacteria (SOB). Location of biofilm: bottom (a), middle (b), top (c) and outdoor (d). Figure S4. Phylogenetic affiliation of phylotypes identified as Bacteroidetes from each biofilm: top pipe (TP, gray) and bottom pipe (BP, black). Clones were identified by genus or order (*) and percentage of each representative sequence in their respective libraries is provided in the brackets. The tree was inferred using maximum likelihood analysis of aligned 16S rRNA gene sequences with bootstrap values from 100 replicates. Box indicates the two most dominant phylotypes. Figure S5. Phylogenetic affiliation of Deltaproteobacteria phylotypes identified as sulfate-reducing bacteria (SRB) from each biofilm: top pipe (TP, gray) and bottom pipe (BP, black). Clones were identified by genus or family (*) and percentage of each representative sequence in their respective libraries is provided in the brackets. The tree [file 1471-2180-12-122-S1.pdf]

Figure S1

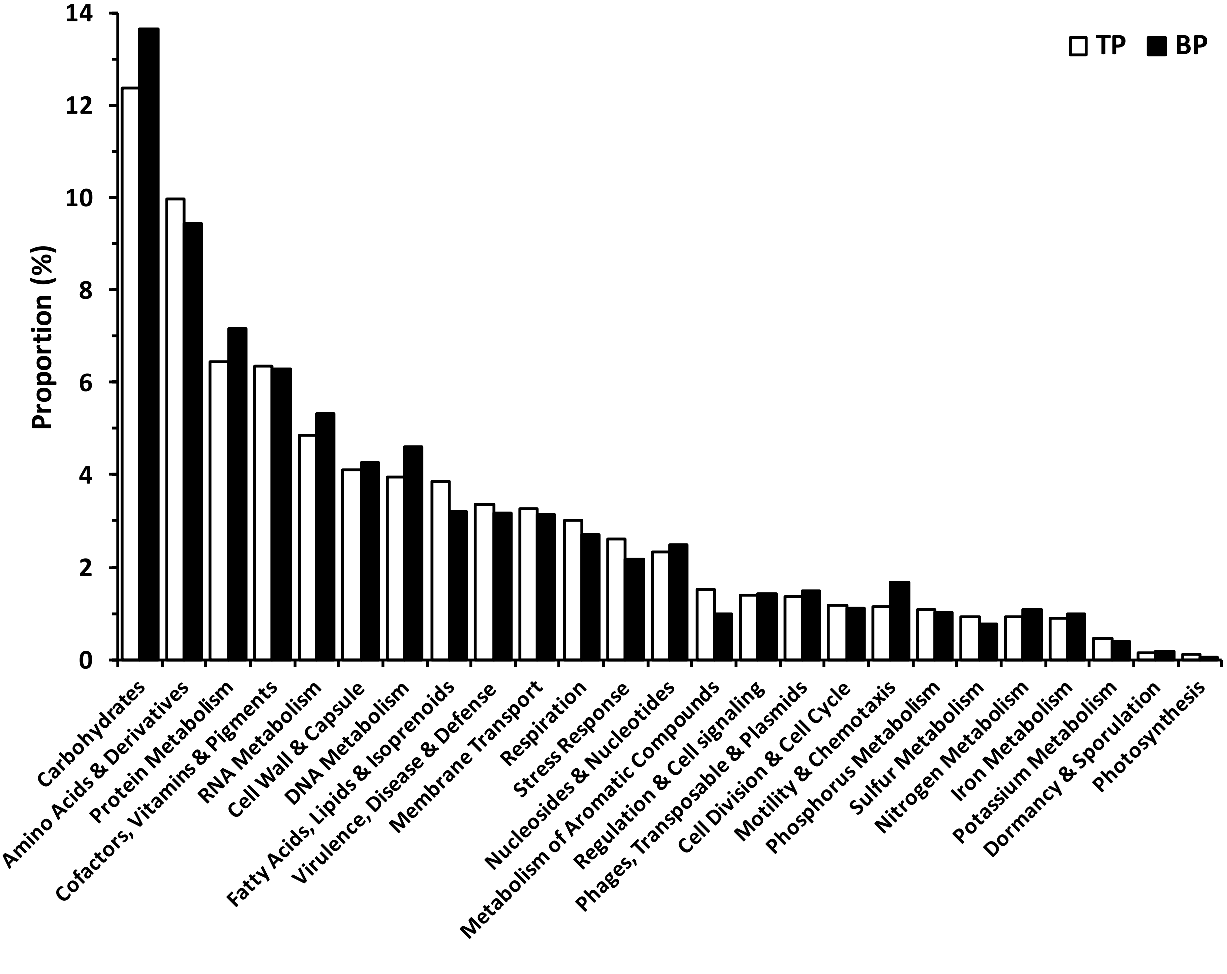

TP
BP
Proportion (%)
14
12
10
8
6
4
2
0
Carbohydrates
Amino Acids & Derivatives
Protein Metabolism
Cofactors, Vitamins & Pigments
RNA Metabolism
Cell Wall & Capsule
DNA Metabolism
Fatty Acids, Lipids & Isoprenoids
Virulence, Disease & Defense
Membrane Transport
Respiration
Stress Response
Nucleosides & Nucleotides
Metabolism of Aromatic Compounds
Regulation & Cell signaling
Phages, Transposable & Plasmids
Cell Division & Cell Cycle
Motility & Chemotaxis
Phosphorus Metabolism
Sulfur Metabolism
Nitrogen Metabolism
Iron Metabolism
Potassium Metabolism
Dormancy & Sporulation
Photosynthesis

Figure S2

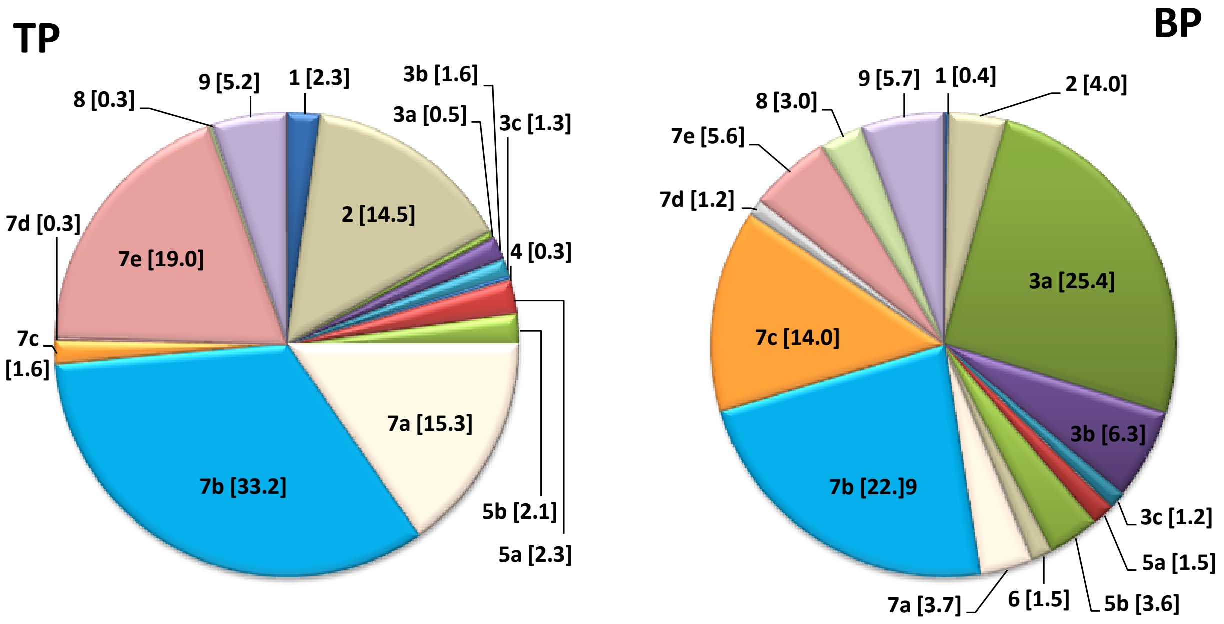
TP
9 [5.2]
1 [2.3]
3b [1.6]
8 [0.3]
3a [0.5]
3c [1.3]
2 [14.5]
7d [0.3]
4 [0.3]
7e [19.0]
7c
[1.6]
7a [15.3]
7b [33.2]
5b [2.1]
5a [2.3]
BP
9 [5.7]
1 [0.4]
2 [4.0]
8 [3.0]
7e [5.6]
7d [1.2]
3a [25.4]
7c [14.0]
3b [6.3]
7b [22.]9
3c [1.2]
5a [1.5]
7a [3.7]
6 [1.5]
5b [3.6]

Figure S3

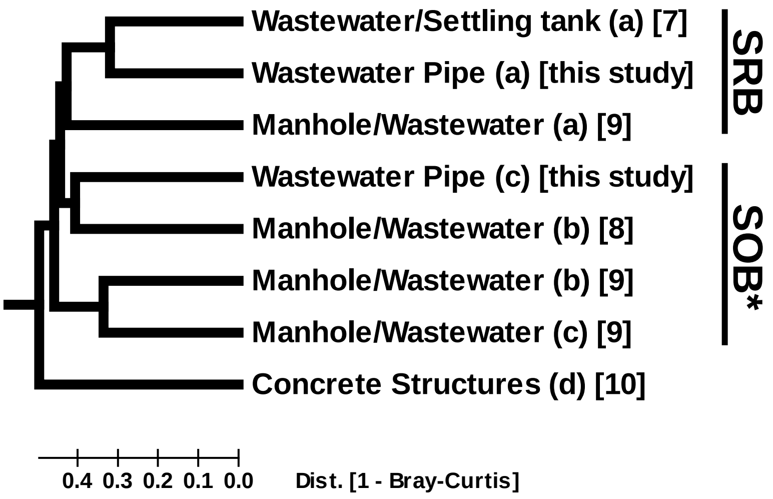
Wastewater/Settling tank (a) [7]
Wastewater Pipe (a) [this study]
Manhole/Wastewater (a) [9]
Wastewater Pipe (c) [this study]
Manhole/Wastewater (b) [8]
Manhole/Wastewater (b) [9]
Manhole/Wastewater (c) [9]
Concrete Structures (d) [10]
SRB
SOB*
0.4 0.3 0.2 0.1 0.0
Dist. [1 - Bray-Curtis]

Figure S4

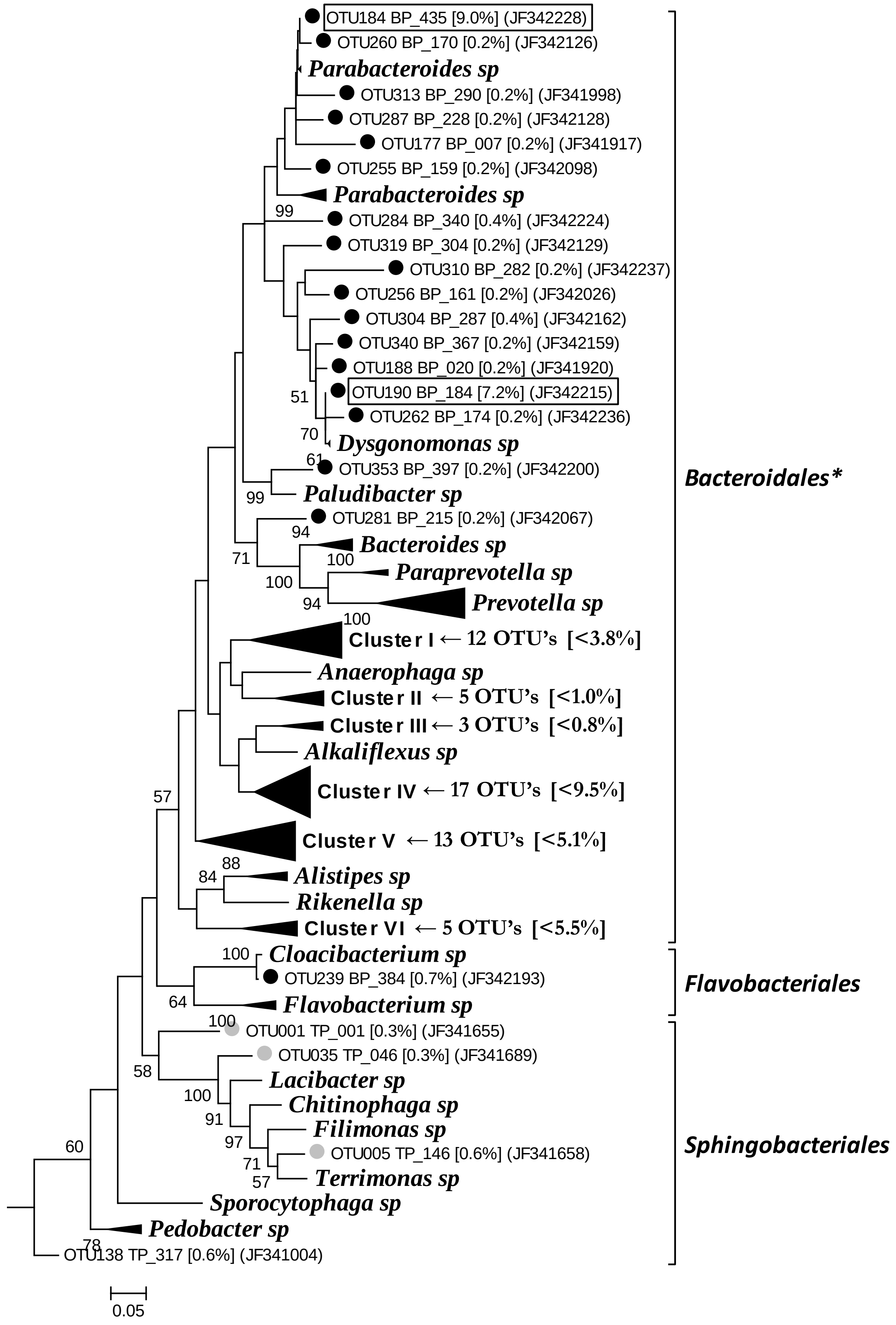

OTU184 BP_435 [9.0%] (JF342228)
OTU260 BP_170 [0.2%] (JF342126)
Parabacteroides sp
OTU313 BP_290 [0.2%] (JF341998)
OTU287 BP_228 [0.2%] (JF342128)
OTU177 BP_007 [0.2%] (JF341917)
OTU255 BP_159 [0.2%] (JF342098)
Parabacteroides sp
99
OTU284 BP_340 [0.4%] (JF342224)
OTU319 BP_304 [0.2%] (JF342129)
OTU310 BP_282 [0.2%] (JF342237)
OTU256 BP_161 [0.2%] (JF342026)
OTU304 BP_287 [0.4%] (JF342162)
OTU340 BP_367 [0.2%] (JF342159)
OTU188 BP_020 [0.2%] (JF341920)
51
OTU190 BP_184 [7.2%] (JF342215)
OTU262 BP_174 [0.2%] (JF342236)
70
Dysgonomonas sp
61
OTU353 BP_397 [0.2%] (JF342200)
99
Paludibacter sp
OTU281 BP_215 [0.2%] (JF342067)
94
Bacteroides sp
71
100
Paraprevotella sp
100
94
Prevotella sp
100
Cluster I ← 12 OTU's [<3.8%]
Anaerophaga sp
Cluster II ← 5 OTU's [<1.0%]
Cluster III ← 3 OTU's [<0.8%]
Alkaliflexus sp
Cluster IV ← 17 OTU's [<9.5%]
57
Cluster V ← 13 OTU's [<5.1%]
88
84
Alistipes sp
Rikenella sp
Cluster VI ← 5 OTU's [<5.5%]
100
Cloacibacterium sp
OTU239 BP_384 [0.7%] (JF342193)
64
Flavobacterium sp
100
OTU001 TP_001 [0.3%] (JF341655)
OTU035 TP_046 [0.3%] (JF341689)
58
Lacibacter sp
100
Chitinophaga sp
91
Filimonas sp
97
OTU005 TP_146 [0.6%] (JF341658)
71
57
Terrimonas sp
60
Sporocytophaga sp
Pedobacter sp
78
OTU138 TP_317 [0.6%] (JF341004)
0.05
Bacteroidales*
Flavobacteriales
Sphingobacteriales

Figure S5

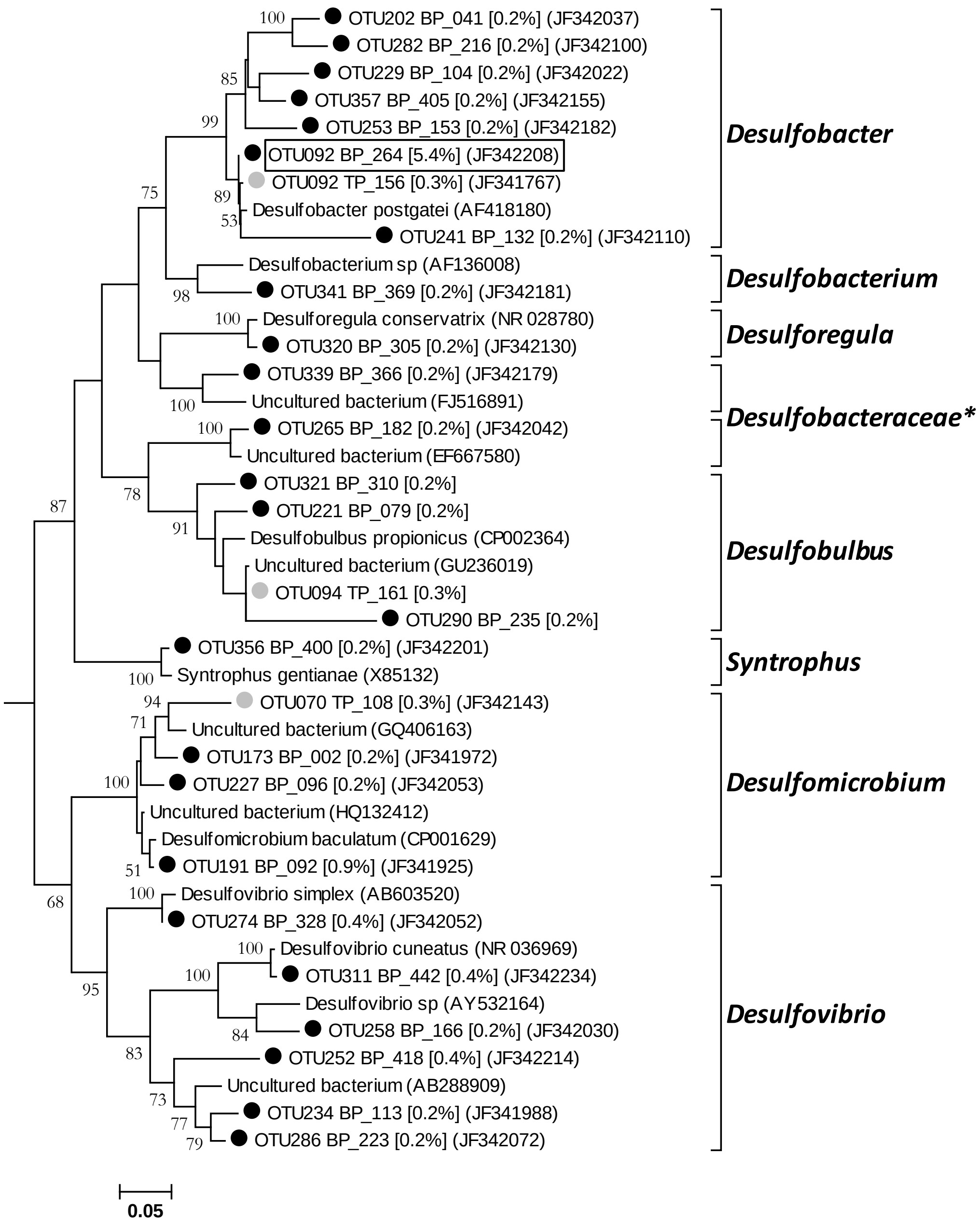

OTU202 BP_041 [0.2%] (JF342037)
OTU282 BP_216 [0.2%] (JF342100)
OTU229 BP_104 [0.2%] (JF342022)
OTU357 BP_405 [0.2%] (JF342155)
OTU253 BP_153 [0.2%] (JF342182)
OTU092 BP_264 [5.4%] (JF342208)
OTU092 TP_156 [0.3%] (JF341767)
Desulfobacter postgatei (AF418180)
OTU241 BP_132 [0.2%] (JF342110)
Desulfobacterium sp (AF136008)
OTU341 BP_369 [0.2%] (JF342181)
Desulforegula conservatrix (NR 028780)
OTU320 BP_305 [0.2%] (JF342130)
OTU339 BP_366 [0.2%] (JF342179)
Uncultured bacterium (FJ516891)
OTU265 BP_182 [0.2%] (JF342042)
Uncultured bacterium (EF667580)
OTU321 BP_310 [0.2%]
OTU221 BP_079 [0.2%]
Desulfobulbus propionicus (CP002364)
Uncultured bacterium (GU236019)
OTU094 TP_161 [0.3%]
OTU290 BP_235 [0.2%]
OTU356 BP_400 [0.2%] (JF342201)
Syntrophus gentianae (X85132)
OTU070 TP_108 [0.3%] (JF342143)
Uncultured bacterium (GQ406163)
OTU173 BP_002 [0.2%] (JF341972)
OTU227 BP_096 [0.2%] (JF342053)
Uncultured bacterium (HQ132412)
Desulfomicrobium baculatum (CP001629)
OTU191 BP_092 [0.9%] (JF341925)
Desulfovibrio simplex (AB603520)
OTU274 BP_328 [0.4%] (JF342052)
Desulfovibrio cuneatus (NR 036969)
OTU311 BP_442 [0.4%] (JF342234)
Desulfovibrio sp (AY532164)
OTU258 BP_166 [0.2%] (JF342030)
OTU252 BP_418 [0.4%] (JF342214)
Uncultured bacterium (AB288909)
OTU234 BP_113 [0.2%] (JF341988)
OTU286 BP_223 [0.2%] (JF342072)
Desulfobacter
Desulfobacterium
Desulforegula
Desulfobacteraceae*
Desulfobulbus
Syntrophus
Desulfomicrobium
Desulfovibrio
0.05

Figure S6

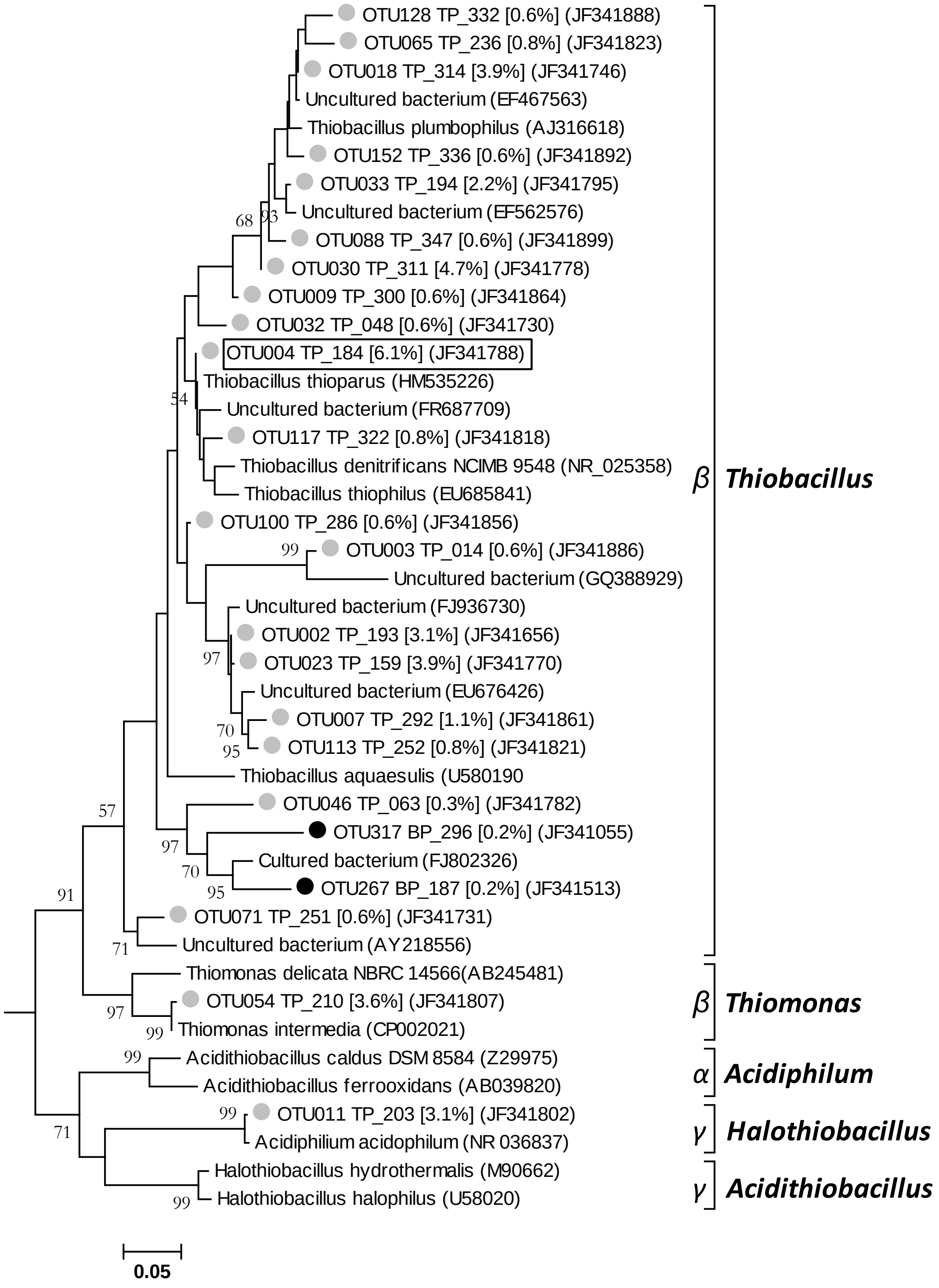

OTU128 TP_332 [0.6%] (JF341888)
OTU065 TP_236 [0.8%] (JF341823)
OTU018 TP_314 [3.9%] (JF341746)
Uncultured bacterium (EF467563)
Thiobacillus plumbophilus (AJ316618)
OTU152 TP_336 [0.6%] (JF341892)
OTU033 TP_194 [2.2%] (JF341795)
Uncultured bacterium (EF562576)
OTU088 TP_347 [0.6%] (JF341899)
OTU030 TP_311 [4.7%] (JF341778)
OTU009 TP_300 [0.6%] (JF341864)
OTU032 TP_048 [0.6%] (JF341730)
OTU004 TP_184 [6.1%] (JF341788)
Thiobacillus thioparus (HM535226)
Uncultured bacterium (FR687709)
OTU117 TP_322 [0.8%] (JF341818)
Thiobacillus denitrificans NCIMB 9548 (NR_025358)
Thiobacillus thiophilus (EU685841)
OTU100 TP_286 [0.6%] (JF341856)
OTU003 TP_014 [0.6%] (JF341886)
Uncultured bacterium (GQ388929)
Uncultured bacterium (FJ936730)
OTU002 TP_193 [3.1%] (JF341656)
OTU023 TP_159 [3.9%] (JF341770)
Uncultured bacterium (EU676426)
OTU007 TP_292 [1.1%] (JF341861)
OTU113 TP_252 [0.8%] (JF341821)
Thiobacillus aquaesulis (U580190)
OTU046 TP_063 [0.3%] (JF341782)
OTU317 BP_296 [0.2%] (JF341055)
Cultured bacterium (FJ802326)
OTU267 BP_187 [0.2%] (JF341513)
OTU071 TP_251 [0.6%] (JF341731)
Uncultured bacterium (AY218556)
Thiomonas delicata NBRC 14566(AB245481)
OTU054 TP_210 [3.6%] (JF341807)
Thiomonas intermedia (CP002021)
Acidithiobacillus caldus DSM 8584 (Z29975)
Acidithiobacillus ferrooxidans (AB039820)
OTU011 TP_203 [3.1%] (JF341802)
Acidiphilium acidophilum (NR 036837)
Halothiobacillus hydrothermalis (M90662)
Halothiobacillus halophilus (U58020)
68
93
54
99
97
70
95
57
97
70
95
91
71
97
99
99
99
71
99
β Thiobacillus
β Thiomonas
α Acidiphilum
γ Halothiobacillus
γ Acidithiobacillus
0.05

Figure S7

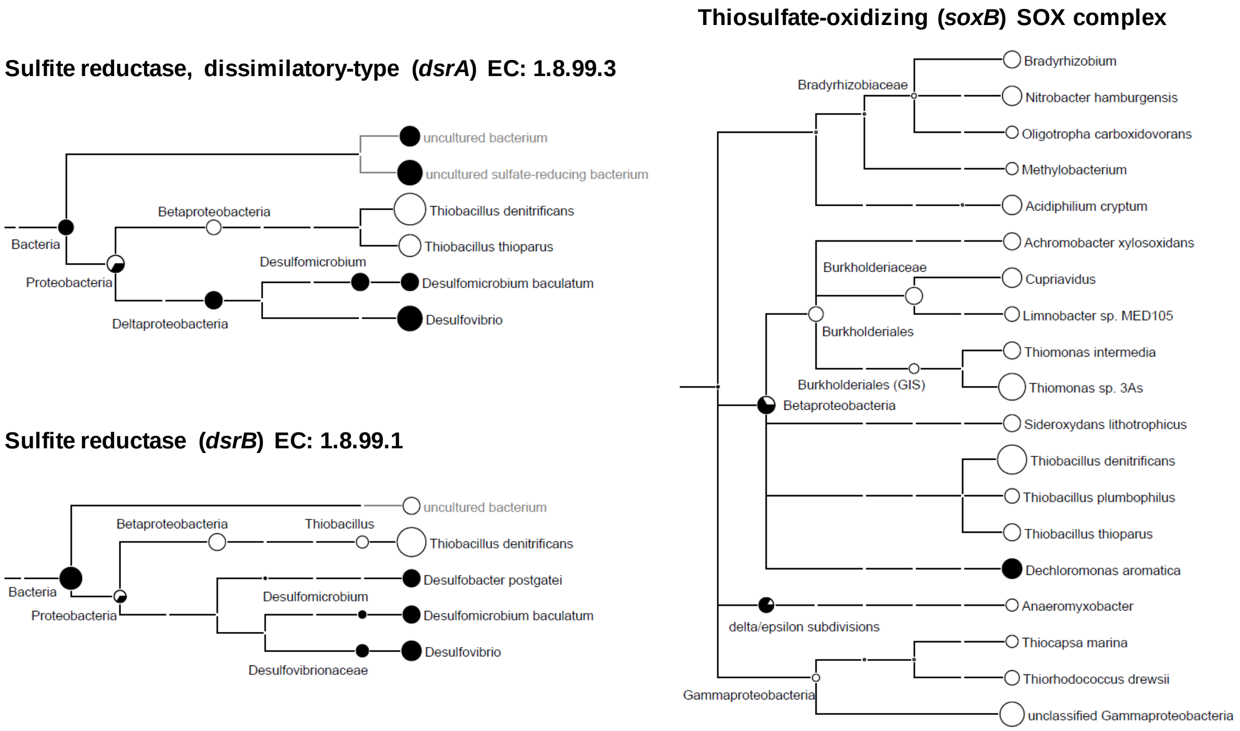

Sulfite reductase, dissimilatory-type (dsrA) EC: 1.8.99.3
uncultured bacterium
uncultured sulfate-reducing bacterium
Betaproteobacteria
Thiobacillus denitrificans
Bacteria
Thiobacillus thioparus
Desulfomicrobium
Proteobacteria
Desulfomicrobium baculatum
Deltaproteobacteria
Desulfovibrio
Sulfite reductase (dsrB) EC: 1.8.99.1
uncultured bacterium
Betaproteobacteria
Thiobacillus
Thiobacillus denitrificans
Desulfobacter postgatei
Bacteria
Desulfomicrobium
Proteobacteria
Desulfomicrobium baculatum
Desulfovibrio
Desulfovibrionaceae
Thiosulfate-oxidizing (soxB) SOX complex
Bradyrhizobium
Bradyrhizobiaceae
Nitrobacter hamburgensis
Oligotropha carboxidovorans
Methylobacterium
Acidiphilium cryptum
Achromobacter xylosoxidans
Burkholderiaceae
Cupriavidus
Limnobacter sp. MED105
Burkholderiales
Thiomonas intermedia
Burkholderiales (GIS)
Thiomonas sp. 3As
Betaproteobacteria
Sideroxydans lithotrophicus
Thiobacillus denitrificans
Thiobacillus plumbophilus
Thiobacillus thioparus
Dechloromonas aromatica
Anaeromyxobacter
delta/epsilon subdivisions
Thiocapsa marina
Thiorhodococcus drewsii
Gammaproteobacteria
unclassified Gammaproteobacteria

Figure S8

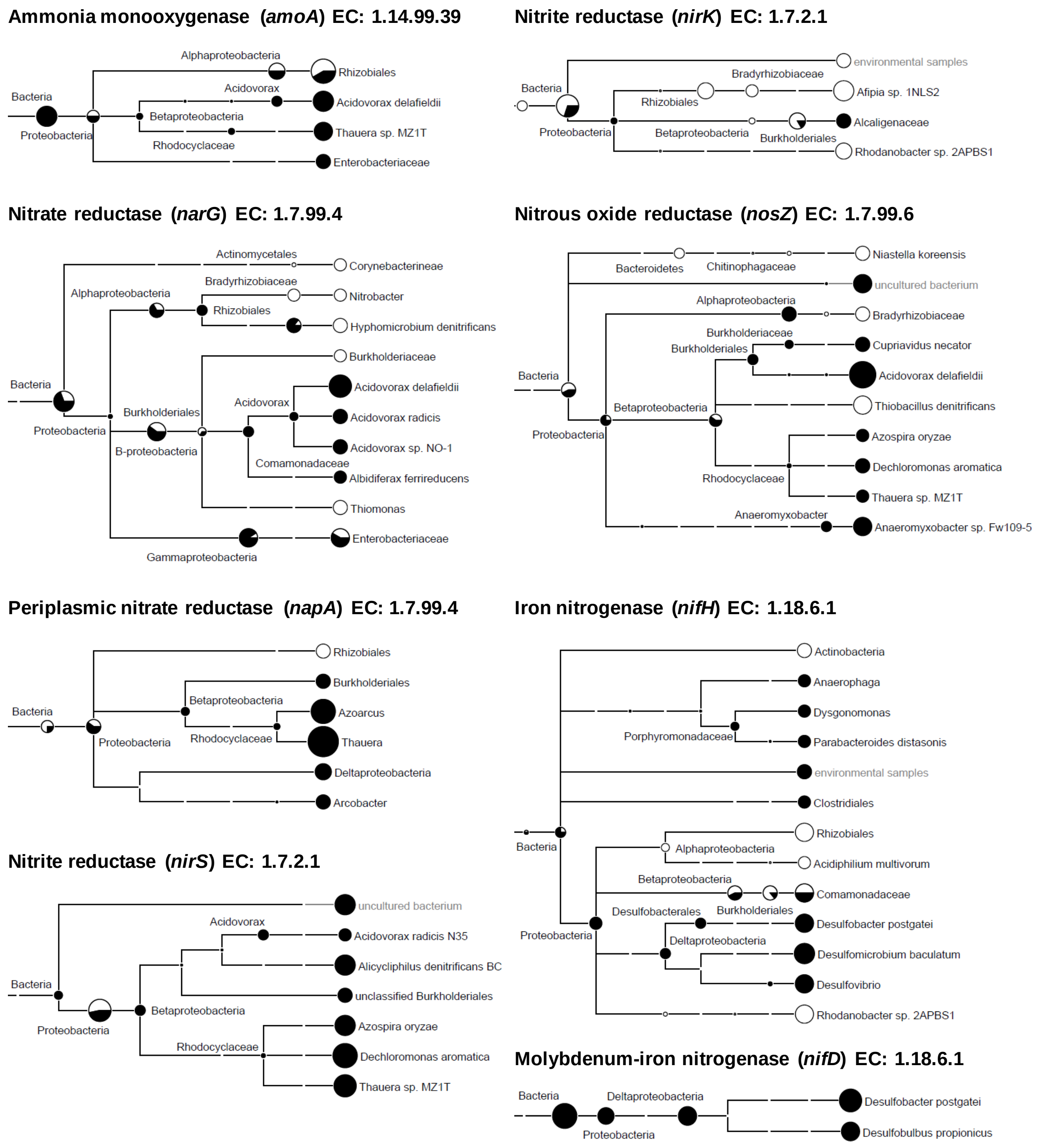
Ammonia monooxygenase (amoA) EC: 1.14.99.39
Bacteria
Proteobacteria
Alphaproteobacteria
Rhizobiales
Acidovorax
Acidovorax delafieldii
Betaproteobacteria
Thauera sp. MZ1T
Rhodocyclaceae
Enterobacteriaceae
Nitrite reductase (nirK) EC: 1.7.2.1
environmental samples
Bradyrhizobiaceae
Bacteria
Rhizobiales
Afipia sp. 1NLS2
Alcaligenaceae
Proteobacteria
Betaproteobacteria
Burkholderiales
Rhodanobacter sp. 2APBS1
Nitrate reductase (narG) EC: 1.7.99.4
Actinomycetales
Corynebacterineae
Bradyrhizobiaceae
Alphaproteobacteria
Nitrobacter
Rhizobiales
Hyphomicrobium denitrificans
Burkholderiaceae
Bacteria
Acidovorax delafieldii
Acidovorax
Burkholderiales
Acidovorax radicis
Proteobacteria
B-proteobacteria
Acidovorax sp. NO-1
Comamonadaceae
Albidiferax ferrireducens
Thiomonas
Enterobacteriaceae
Gammaproteobacteria
Nitrous oxide reductase (nosZ) EC: 1.7.99.6
Niastella koreensis
Bacteroidetes
Chitinophagaceae
uncultured bacterium
Alphaproteobacteria
Bradyrhizobiaceae
Burkholderiaceae
Burkholderiales
Cupriavidus necator
Bacteria
Acidovorax delafieldii
Betaproteobacteria
Thiobacillus denitrificans
Proteobacteria
Azospira oryzae
Rhodocyclaceae
Dechloromonas aromatica
Thauera sp. MZ1T
Anaeromyxobacter
Anaeromyxobacter sp. Fw109-5
Periplasmic nitrate reductase (napA) EC: 1.7.99.4
Rhizobiales
Burkholderiales
Betaproteobacteria
Bacteria
Azoarcus
Proteobacteria
Rhodocyclaceae
Thauera
Deltaproteobacteria
Arcobacter
Nitrite reductase (nirS) EC: 1.7.2.1
uncultured bacterium
Acidovorax
Acidovorax radicis N35
Alicycliphilus denitrificans BC
Bacteria
unclassified Burkholderiales
Betaproteobacteria
Proteobacteria
Azospira oryzae
Rhodocyclaceae
Dechloromonas aromatica
Thauera sp. MZ1T
Iron nitrogenase (nifH) EC: 1.18.6.1
Actinobacteria
Anaerophaga
Dysgonomonas
Porphyromonadaceae
Parabacteroides distasonis
environmental samples
Clostridiales
Bacteria
Rhizobiales
Alphaproteobacteria
Acidiphilium multivorum
Betaproteobacteria
Comamonadaceae
Desulfobacterales
Burkholderiales
Proteobacteria
Desulfobacter postgatei
Deltaproteobacteria
Desulfomicrobium baculatum
Desulfovibrio
Rhodanobacter sp. 2APBS1
Molybdenum-iron nitrogenase (nifD) EC: 1.18.6.1
Bacteria
Deltaproteobacteria
Desulfobacter postgatei
Proteobacteria
Desulfobulbus propionicus
